# The Anthropometric Measurement of Schober's Test in Normal Taiwanese Population

**DOI:** 10.1155/2015/256365

**Published:** 2015-07-27

**Authors:** Yong-Ren Yen, Jin-Fan Luo, Ming-Li Liu, Fung-Jou Lu, Soo-Ray Wang

**Affiliations:** ^1^Institute of Medicine, Chung Shan Medical University, Taichung 40201, Taiwan; ^2^Taichung Branch, Bureau of Standards, Metrology and Inspection (BSMI), M.O.E.A., Taiwan; ^3^Department of Rehabilitation, Jen-Teh Junior College of Medicine, Nursing and Management, Miaoli 35664, Taiwan; ^4^Department of Internal Medicine, Chung Shan Medical University Hospital, Taichung 40201, Taiwan

## Abstract

The measurement of lower back mobility is essential in the assessment of lower back pain including ankylosing spondylitis. Original Schober's test (OST) and modified Schober's test (MST) are popularly conducted in daily rheumatology and orthopedics clinical practices. To our knowledge, this report is the only anthropometric reference study in a normal oriental population. The OST declined with age from 5.0 cm in the youngest (20–30 years old) to 3.1 cm in the aged (70–80 years old) male subjects and from 3.6 cm to 2.4 cm in the female subjects. The male OST was significantly more than the female OST. There was a good correlation between OST and MST in each of the three age groups of both sexes.

## 1. Introduction

The mobility of the lumbar spine is restricted in patients with ankylosing spondylitis (AS) [1]. AS is a chronic inflammatory autoimmune disease of the axial skeleton primarily involving spine and sacroiliac joints [2, 3]. The typical early symptoms are chronic low back pain and spinal stiffness causing difficulty in bending [4]. Eventually it results in complete fusion and rigidity of spinal joints [5]. The most simple and noninvasive screening method for lumbar mobility is Schober's test.

Schober's tests, including original Schober's test (OST) [6–8], modified Schober's test (MST) [7, 8], and modified-modified Schober's test (MMST) [7, 9], are not harmful for measuring flexibility of lumbar spine with the subject bending forward. These tests can be simply conducted in the clinic. The OST and MST are popularly used [7, 10–13]. The measurement of these tests is not only useful for screening the status of AS disease, but also useful for the determination of progression and therapeutic effects of AS [14] as well as other pathologic conditions associated with low back pain [7, 10, 13, 15].

Reports of anthropometric reference data are rare [11, 16], and none were reported in oriental populations. We measured the Schober indices in different ages of normal Taiwanese men and women who had no history of low back pain or any diseases involving the lower back. This study provides reference data for clinicians to judge the degree of low back motility and disease status [16]. We measured OST in normal subjects for lumbar mobility in relation to age and sex. The correlation between OST and MST was also studied.

## 2. Methods and Materials

Volunteer subjects were recruited from the clinic of AIR (allergy, immunology, and rheumatology), Chung Shan Medical University Hospital in central Taiwan. The exclusion criteria included any structural abnormality such as kyphosis, scoliosis, surgical history, lumbar deformity, and skin problems of the lumbar area and functional abnormality such as arthropathy, acute or chronic pain in the lumbar area, or neurological diseases involving the lumbar area.

There were 165 men and 122 women for OST measurements and 135 men and 84 women for both OST and MST measurements.

The OST and MST were measured according to the methods described [8]. Namely, for the measurement of OST, the participant stood erect while the lumbosacral junction was marked as indicated by the dimples of Venus. A second mark was placed 10 cm above the junction. The participant was then asked to bend forward as far as possible, and the stretched distance was indicated as the OST in cm.

For measuring the MST, we put a mark 5 cm below and 10 cm above the junction. The participant was asked to bend forward as far as possible and the stretched distance of these two points was measured as the MST value.

Statistical analysis of the differences between different age and sex groups was carried out using the Statistical Package for the Social Sciences (SPSS), version 7.0 (Chicago, IL, USA). A value of *P* < 0.05 was considered significant.

The design of this study about the measurement of lower back flexibility conformed to the Declaration of Helsinki and this project was reviewed and approved by the Institutional Review Board of the Chung Medical University Hospital (CSMUH number 13211).

## 3. Results

### 3.1. The OST among Different Age Groups

**Figure 1:**
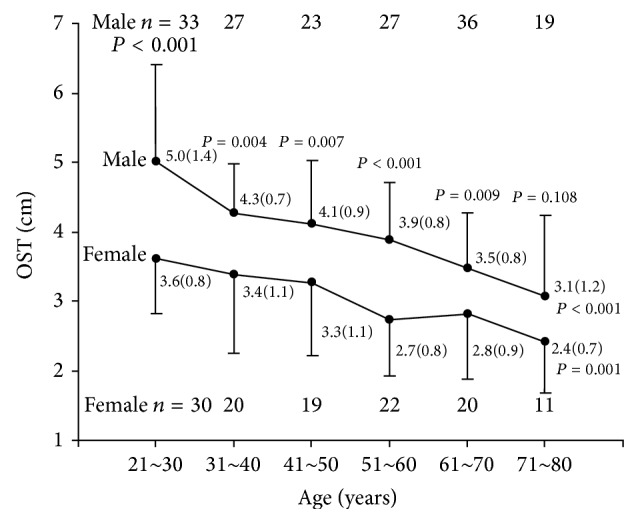
The relation between original Schober's test (OST) and age. OST data were presented as the mean (±SD).

For the measurement of OST, 165 men and 122 women were recruited. The OST measurements in different age groups and in both sexes are shown in Figure 1.

The OST declined with age as tested by ANOVA, *P* < 0.001 for men and *P* = 0.001 for women. For male subjects, OST decreased an average of 0.38 cm for each decade of age and 0.24 cm for female subjects. The male OST, by *t*-test, was significantly more than the female OST in each age group (*P* values were between 0.001 and 0.009) except for those older than 71 years of age. On average, the male OST was 0.95 cm more than the female OST.

### 3.2. The Relationship between OST and MST

**Figure 2:**
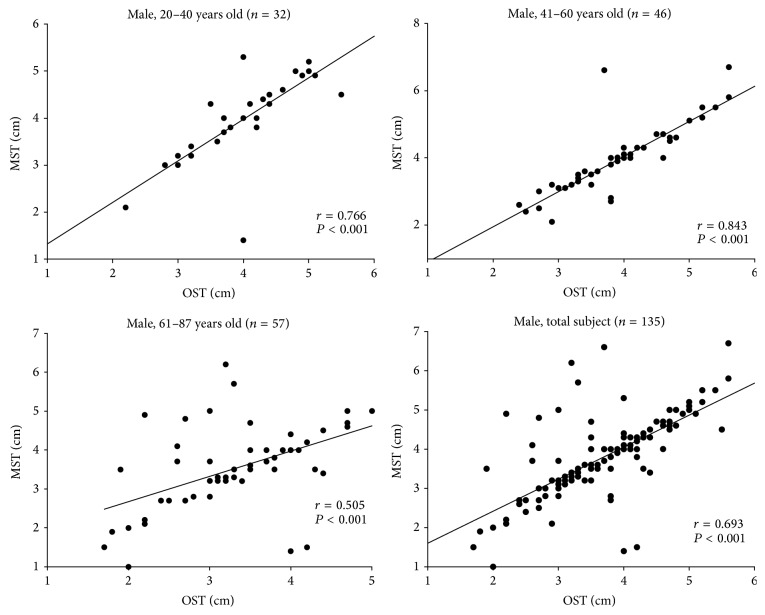
The correlation between original Schober's test (OST) and modified Schober's test (MST) in the male group.

In this study, all subjects (*n* = 219) were divided into three age groups, 20–40, 41–60, and above 60 years old. For males (*n* = 135) (Figure 2), there was a close correlation between OST and MST in all three age groups and total male subjects with *P* < 0.001 by a Pearson correlation test. In each panel, the coefficient of correlation, *r*, was between 0.505 and 0.843.

**Figure 3:**
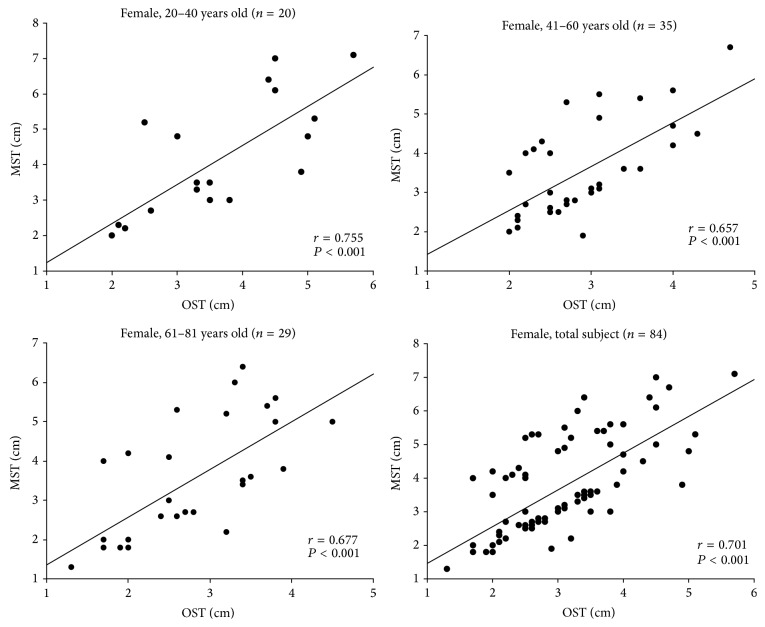
The correlation between original Schober's test (OST) and modified Schober's test (MST) in the female group.

For females (*n* = 84) (Figure 3), there was a good correlation between OST and MST with *P* < 0.001 for all three age groups and total female subjects. In each panel, the *r* was between 0.657 and 0.755. These data seemed more scattered than in the male group.

## 4. Discussion

To our knowledge, this is the first report on anthropometric measurements in a normal oriental population.

Our data showed that both the OST and the MST declined with age, there being more lumbar mobility in the younger subjects than the aged (Figure 1). The degree of mobility declination of the OST over age among males was very significant from an average of 5.0 cm in the youngest group to 3.1 cm in the oldest group and 3.6 cm to 2.4 cm among females. It was difficult to recruit enough aged normal subjects for this study. We recruited only 11 normal female subjects aged 71–80 in this study, making the results less reliable. The lumbar mobility in males was significantly more than the lumbar mobility among females, being 0.95 cm more on average.

Schober's test results were partitioned into OST, MST, and MMST [7]. Each test is easy to perform in the clinic. Our results indicated that there was a good correlation between the OST and MST results in all the three age groups, *P* < 0.001 (Figures 2 and 3). Thus, one can select either test to perform in daily clinic practice.

According to the report of Cidem et al. [8], the factor of body height was not statistically significant in correlation with OST results; hence we did not measure body heights in our study subjects. In their study in a normal male Turkish population [8], the OST was 5.6 cm in a young male group (ages 20–30), while it was 5.0 cm in the same age group of the normal Taiwanese subjects. It seemed to be not due to the difference of body height in the two races but rather maybe due to hereditary or life style differences, such as exercise.

The lumbar mobility is much less in patients with ankylosing spondylitis (AS). Tan et al. [12] reported that the OST of AS patients with disease duration of 20.0 ± 11.8 years was 3.3 ± 1.2 cm, which is equivalent to our study at ages between 60 and 80 years. In Inanir's report [17], in 48 AS patients with normal heart, having no cardiac disorder of fragmented QRS, the OST was 4.10 ± 1.40 cm. Rezvani et al. [7] reported that the mean OST of AS patients was 4.07 ± 1.88 cm, while the control was 4.57 ± 0.87 cm, being 12.3% more than that of AS patients. The OST of their control is equivalent to that of our report at ages between 21 and 40 years. In patients with acute low back pain, the OST was even less, being only 2 cm, as reported by Konstantinovic et al. [18].

In our study, the subjects with pathological lumbar conditions were strictly excluded, such as functional abnormality, structural abnormality, and lumbar skin problems. The data presented here measuring the normal subjects is purely aimed as reference data to be used for comparison with various diseases in oriental subjects.

## 5. Conclusion

The anthropometric measurements of Schober's test (OST) in a Taiwanese population showed significant declination with age. The male OST values were significantly more than the OST values in females. There was a good correlation between OST and MST in each of three age groups and between sexes.
